# Prognostic Value of Preoperative Hemoglobin Levels for Long-Term Outcomes of Acute Type B Aortic Dissection Post-thoracic Endovascular Aortic Repair

**DOI:** 10.3389/fcvm.2020.588761

**Published:** 2020-11-05

**Authors:** Zhichun Gao, Zhexue Qin, Zhixia An, Changchun Hou, Luyu Wang, Jun Jin

**Affiliations:** ^1^Department of Cardiology, Institute of Cardiovascular Diseases of People's Liberation Army, Chongqing, China; ^2^Department of Cardiology, Xinqiao Hospital, Army Medical University (Third Military Medical University), Chongqing, China

**Keywords:** hemoglobin, thoracic endovascular aortic repair, prognosis, aortic dissection, long-term

## Abstract

**Background and Aims:** There is scant information available about the prognostic value of preoperative hemoglobin (Hb) levels on the long-term outcomes of acute type B aortic dissection (ABAD) following thoracic endovascular aortic repair (TEVAR).

**Methods:** A retrospective analysis of consecutive patients from 2010 to 2018 regarding the relationship between Hb level and long-term outcomes was conducted. The primary endpoint was all-cause mortality. Major adverse cardiovascular events (MACEs) included all-cause death, recurrent ruptures, and secondary procedures.

**Results:** In total, 391 subjects treated by TEVAR were enrolled, with a mean age of 57.1 ± 12.0 years; 79.5% of them were male. Cox multivariate analysis showed that the preoperative Hb level was independently associated with all-cause death [adjusted hazard ratio (HR) 0.797 (per 1 g/dl), 95% confidence interval (CI) 0.693–0.918, *p* = 0.002] and MACEs (adjusted HR 0.795, 95% CI 0.672–0.871, *p* = 0.000). The area under the receiver operating characteristic curve of Hb for all-cause death and MACEs were 0.617 (95% CI 0.548–0.687, *p* = 0.008) and 0.617 (95% CI 0.551–0.684, *p* = 0.005), respectively. In the linear trend test, Hb concentration was significantly related to all-cause mortality (*p* for trend = 0.001) and MACEs (*p* for trend = 0.000). Moreover, in Kaplan–Meier analysis, lower Hb levels (< 12 g/dl) were significantly different from higher Hb (≥12 g/dl) levels for both all-cause death (log-rank *p* = 0.001) and MACEs (log-rank *p* = 0.001). Similar results were found when assessing the prognostic value of red blood cell count and anemia.

**Conclusions:** Preoperative Hb may serve as a prognostic marker for long-range adverse outcomes for ABAD patients post-TEVAR.

## Introduction

A dramatic surge in the incidence of acute aortic dissection has occurred over the last decade (1, 2). Aortic dissection is conventionally classified as Stanford A or B, with the latter involving the descending aorta (3). Acute type A aortic dissection is usually treated by surgery, whereas acute type B aortic dissection (ABAD) is often treated by thoracic endovascular aortic repair (TEVAR) and/or optimized drug agents. Despite the introduction of TEVAR, the in-hospital mortality of ABAD remains at 14% (4). According to a recent study (5), the late outcomes for ABAD patients are much worse, even when the TEVAR procedure is performed. Thus, it is of central importance to identify those with a high risk over the long term.

Serological examination has been considered to be a suitable and simple way to assess a patient's status at bedside. For example, the white blood cell (WBC) count, mean platelet volume to platelet count ratio, C-reactive protein (CRP) and serum creatinine levels (6–9) have been reported to be associated with in-hospital outcomes in ABAD subjects. In other studies, N-terminal pro-B-type natriuretic peptide, Th17 cells, hypoalbuminemia, and thrombocytopenia were proposed to predict long-term outcomes of ABAD patients, with a median follow-up period ranging from 2 to 5 years (10–13). However, given the severity and fatality of ABAD, it remains necessary to probe and seek more valuable prognostic indicators.

Hemoglobin (Hb) has been described as a prognostic marker in cardiovascular diseases such as acute heart failure, coronary artery disease, strokes and in those undergoing cardiac surgery (14–17). Yet information about the relationship between Hb and ABAD is limited. Gorla et al. suggested that Hb was independently related to in-hospital outcomes of acute aortic syndrome (AAS) post-TEVAR (18), though 2-year outcomes were not independently associated with Hb levels. However, given its relatively small sample, further studies on the relationship between Hb and ABAD outcomes remain meaningful.

The present study aims to examine the association between Hb concentration and the long-term outcomes of ABAD patients who have undergone TEVAR in a relatively large population with a long follow-up period.

## Method

### Study Population

A single-center retrospective study was conducted from May 2010 to December 2018 in Xinqiao Hospital of the Army Medical University (Third Military Medical University) in Chongqing, China. The participants were consecutively enrolled. The inclusion criteria were (1) diagnosed with ABAD by computed tomography (CT) angiography; (2) acute or subacute phase; and (3) TEVAR. Subjects with Marfan syndrome, trauma, iatrogenic injury, intramural hematoma, or past type A aortic dissection were excluded from the study. Those with severe heart failure, malignant cancer, and myocardial infarction were also excluded. Written informed consent were obtained from all the enrolled subjects. The present study conforms to the ethical guidelines of the 1975 Declaration of Helsinki and was approved by the ethics committee of Xinqiao Hospital.

### Procedure and Treatment

All subjects received optimal antihypertensive therapy using angiotensin-converting enzyme inhibitors (ACEIs) or angiotensin receptor blockers (ARBs), calcium channel blockers, and/or β-blockers. The indication for TEVAR was assessed by multidisciplinary consultation, including two senior cardiologists and one radiologist. TEVAR was performed under general or local anesthesia by percutaneous or open femoral access. A pigtail catheter was advanced for angiography from the contralateral femoral side, with a stent graft delivered over a guide wire. Completion angiography was used to detect type I endoleak, a complication warranting urgent treatment.

### Data Collection and Laboratory Measurements

The demographics and medical history of the patients were extracted from medical records. In this retrospective study, the old diagnostic criterion for hypertension was adopted (19). Diabetes mellitus was validated if fasting glucose > 126 mg/dl or hypoglycemic medications were used. Dyslipidemia was defined as serum total cholesterol higher than 5.72 mmol/L or treatment with antihyperlipidemic drugs. Anemia was defined as Hb level < 12 g/dl for men and < 11 g/dl for women according to Chinese criteria (20). WBC count, red blood cell (RBC), Hb level, serum creatinine, and other serum parameters were measured at admission while fasting and abstaining from smoking. Serum samples for blood cell counts were collected in sealed vacuum tubes from veins with the skin free of burns, edema, or cyanosis; the blood was tested 30 min to 8 h post-ethylenediaminetetraacetic acid anticoagulant processing. The results were obtained using automatic blood analyzers.

### Follow-Up and Endpoint

The participants were followed up by telephone interviews and medical records. If the telephone number was not answered, questionnaires were sent to the address on file at the hospital. Loss to follow-up was defined as no response to three attempts of telephone contact or the questionnaire. To reassure the authenticity of follow-up, 10% of the follow-up results were randomly selected for review. The primary endpoint was defined as all-cause death, and the major adverse cardiovascular event (MACE) was a composite of all-cause death, recurrent rupture of dissection [*International Classification of Diseases, Tenth Revision* (ICD-10), I71.001], and secondary procedure.

### Statistical Analysis

Continuous and categorical variables are described as means ± standard variation and percentages (or frequencies), respectively. Intergroup differences for continuous data were estimated by the two-sample *t*-test or non-parametric Mann–Whitney U test; the Fisher exact or χ^2^ test was utilized to assess intergroup differences for categorical data. The time-to-event curve was assessed by the Kaplan–Meier method, with log-rank tests to compare different curves. Multivariable Cox proportional-hazards regression analysis using a forward conditional method was applied to identify the association between outcomes and Hb levels or RBC, introducing variables with *p* < 0.10 into univariate analysis. The hazard ratio (HR) and 95% confidence intervals (CIs) for Hb in the Cox model indicating the risk difference were calculated by increasing per 1 g/dl. A linear trend test was performed by entering the median value for each category of Hb or RBC quintiles, with HRs and 95% CIs calculated by Cox proportional-hazards regression models using a forward conditional method, which were adjusted for age, chronic obstructive pulmonary disease (COPD), hematocrit, cystatin C, urea nitrogen, and low-density lipoprotein-cholesterol (LDL-C) in the all-cause death model. The MACE model was adjusted for age, COPD, hematocrit, urea nitrogen, cystatin C, creatinine, total cholesterol, and LDL-C. We assessed the effect of Hb and RBC on patient outcomes by an area under the receiver operating characteristic (ROC) curve (AUC). HR and 95% CI were provided to display the power of associations. A two-sided *p* < 0.05 was considered statistically significant. All statistical analyses were conducted using Statistical Package for the Social Sciences version 25.0 (SPSS Inc., Chicago, IL, USA).

## Results

### Demographic and Laboratory Examination Characteristics

In total, 445 patients were initially diagnosed with ABAD and underwent TEVAR (Supplementary Figure 1): 21 patients with traumatic dissections, seven with prior Stanford A dissections, six with Marfan syndrome, and one with iatrogenic injury were excluded. Fifteen subjects with severe heart failure, malignant cancer, or myocardial infarction were also excluded. Furthermore, five patients were lost to follow-up. Consequently, a total of 391 subjects were enrolled in the study. The baseline characteristics are summarized in Table 1. The mean age was 57.1 ± 12.0 years, and 79.5% of the subjects were male. The median follow-up time was 56 months. Current smokers accounted for 54.7% of the included patients; 81.6% of the patients were diagnosed with hypertension. The mean Hb level and RBC count was 12.6 ± 1.9 g/dl and 4.2 ± 0.6 × 10^12^/L, respectively.

**Table 1:** Baseline characteristics at admission of the ABAD patients post-TEVAR.

	**Total**	**Hb < 12 g/dl**	**Hb ≥ 12 g/dl**	***p*-value**
*N*	391	134	257	
Age, years	57.1 ± 12.0	59.4 ± 13.7	56.5 ± 11.0	0.004^*^
Male	311 (79.5)	85 (63.4)	226 (87.9)	0.000^*^
Hypertension	319 (81.6)	105 (78.4)	214 (83.3)	0.234
Diabetes	20 (5.1)	5 (3.7)	15 (5.8)	0.37
Current smoker	214 (54.7)	61 (45.5)	153 (59.5)	0.007^*^
COPD	18 (4.6)	4 (3.0)	14 (5.4)	0.270
Hyperlipidemia	18 (4.6)	0 (0.0)	18 (7.0)	0.002^*^
Drug treatment				
ACEI/ARBs	300 (76.7)	95 (70.9)	205 (79.8)	0.045^*^
CCBs	347 (88.7)	115 (85.8)	232 (90.3)	0.186
β-Blockers	310 (79.3)	100 (74.6)	210 (81.7)	0.085
Diuretics	189 (48.3)	61 (45.5)	128 (49.8)	0.381
SBP, mmHg	147.4 ± 26.3	136.0 ± 22.1	148.0 ± 26.9	0.098
DBP, mmHg	84.7 ± 23.9	85.0 ± 14.9	86.8 ± 54.0	0.046^*^
WBC, ×10^12^/L	9.8 ± 3.6	8.6 ± 3.6	10.0 ± 3.5	0.091
RBC, ×10^12^/L	4.2 ± 0.6	3.5 ± 0.6	4.5 ± 0.5	0.000^*^
Hb, g/dl	12.6 ± 1.9	10.3 ± 1.3	13.7 ± 1.3	0.000^*^
Platelet, ×10^9^/L	167.0 (128.0–226.0)	156.0 (123.0–241.0)	153.0 (122.0–200.0)	0.765
LDH, U/L	197.0 (171.0–237.0)	206.8 (170.0–244.5)	200.5 (173.0–239.0)	0.873
Albumin, g/L	39.7 ± 5.1	37.0 ± 4.3	41.1 ± 4.9	0.000^*^
Cystatin C, mg/L	1.00 (0.82–1.23)	1.05 (0.85–1.44)	0.96 (0.80–1.19)	0.036^*^
Creatinine, μmol/L	78.8 (63.8–98.8)	73.9 (55.5–99)	80.0 (69.0–94.0)	0.934
Urea nitrogen, mmol/L	6.9 ± 3.1	7.4 ± 4.3	6.7 ± 2.9	0.431
LDL-C, mmol/L	2.6 ± 0.8	2.3 ± 0.6	2.7 ± 0.6	0.000^*^
Total cholesterol, mmol/L	4.2 ± 1.1	3.9 ± 1.0	4.4 ± 0.9	0.002^*^

*Continuous values are documented by mean ± SD or median (1st quartile−3rd quartile). Categorical variables are recorded by n (percentage)*.

*ABAD, acute type B aortic dissection; TEVAR, thoracic endovascular aortic repair; CCB, calcium-channel blocker; ACEI, angiotensin-converting enzyme inhibitor; ARB, angiotensin receptor blocker; SBP, systolic blood pressure; DBP, diastolic blood pressure; WBC, white blood (cell) count; RBC, red blood cell count; COPD, chronic obstructive pulmonary disease; Hb, hemoglobin; LDH, lactate dehydrogenase; LDL-C, low-density lipoprotein-cholesterol*.

* *p < 0.05*.

Patients with preoperative Hb levels < 12 g/dl were likely older, female, non-smokers, and without hyperlipidemia. Moreover, they were more likely to have higher cystatin C and lower diastolic blood pressure (DBP), RBC, serum albumin, LDL-C, and total cholesterol levels and less likely be treated with ACEIs/ARBs. Compared with survivors, the deceased were frequently older; had COPD, higher cystatin C, and urea nitrogen; and were more likely to have lower Hb, RBC, platelet, LDL-C, and total cholesterol levels (Table 2).

**Table 2:** Baseline characteristics for survivors and non-survivors.

	**Survivors**	**Non-survivors**	***p*-value**
*N*	304	87	
Age, years	56.2 ± 12.1	60.6 ± 10.9	0.001^*^
Male	236 (77.6)	75 (86.2)	0.080
Current smoker	164 (53.9)	50 (57.5)	0.580
Hypertension	246 (80.9)	73 (83.9)	0.236
Diabetes	18 (5.9)	2 (2.3)	0.269
COPD	10 (3.3)	8 (9.2)	0.038^*^
Hyperlipidemia	17 (5.6)	1 (1.1)	0.141
SBP, mmHg	147.2 ± 26.2	148.5 ± 26.7	0.491
DBP, mmHg	84.8 ± 25.9	84.5 ± 15.0	0.410
WBC, ×10^12^/L	9.8 ± 3.5	9.7 ± 4.0	0.524
RBC, ×10^12^/L	4.3 ± 0.6	4.0 ± 0.6	0.000^*^
Hb, g/dl	12.8 ± 1.9	12.0 ± 1.9	0.000^*^
Platelet, ×10^9^/L	174.0 (134.0–226.3)	146.5 (114.5–229.5)	0.020^*^
LDH, U/L	196.0 (168.3–231.7)	204.3 (179.6–260.6)	0.095
Albumin, g/L	39.9 ± 5.2	39.1 ± 4.7	0.119
Creatinine, μmol/L	78.2 (63.9–96.7)	82.1 (62.7–120.4)	0.124
Cystatin C, mg/L	0.97 (0.80–1.17)	1.09 (0.90–1.49)	0.004^*^
Urea nitrogen, mmol/L	6.7 ± 3.1	7.5 ± 3.1	0.024^*^
LDL-C, mmol/L	2.7 ± 0.8	2.3 ± 0.8	0.000^*^
Total cholesterol, mmol/L	4.3 ± 0.9	3.9 ± 1.5	0.000^*^

*Continuous values are documented by mean ± SD or median (1st quartile−3rd quartile). Categorical variables are recorded by n (percentage)*.

*SBP, systolic blood pressure; DBP, diastolic blood pressure; RBC, red blood cell count; COPD, chronic obstructive pulmonary disease; Hb, hemoglobin; LDH, lactate dehydrogenase; LDL-C, low-density lipoprotein-cholesterol; WBC, white blood (cell) count*.

* *p < 0.05*.

### Overall Outcomes

**Figure 1:**
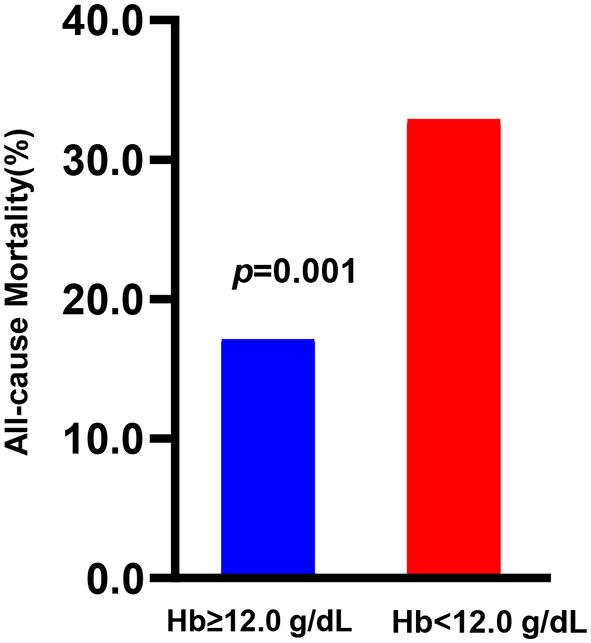
All-cause mortality between different hemoglobin levels.

As shown in Figure 1, outcomes were significantly different in terms of all-cause mortality between the preoperative Hb < 12 g/dl and Hb ≥ 12 g/dl groups (32.1% vs. 17.1%, *p* = 0.001). In contrast, no significant difference in in-hospital mortality (3.73% vs. 2.33%, *p* = 0.428), secondary procedures (2.24% vs. 0.78%, *p* = 0.344), or recurrent rupture (1.49% vs. 1.56%, *p* = 1.000) was found between the two groups (Supplementary Figure 2).

### Area Under the Receiver Operating Characteristic Curves and Linear Trend Test

The AUCs for preoperative Hb regarding all-cause death and MACEs were 0.617 (95% CI 0.548–0.687, *p* = 0.008) and 0.617 (95% CI 0.551–0.684, *p* = 0.005), respectively (Supplementary Figure 3). The cutoff points for preoperative Hb with regard to all-cause death and MACEs were 11.05 and 11.15 g/dl (Supplementary Table 1). The linear trend test results between preoperative Hb concentration and outcomes are depicted in Table 3, and a lower Hb level was significantly associated with a higher risk of all-cause death (*p* for trend = 0.001) and MACEs (*p* for trend = 0.000), despite adjustment for the variables listed in Table 3. The HRs for the lowest quintiles of Hb (with the highest quintiles as reference) were 5.64 (95% CI 1.89–16.87) and 6.15 (95% CI 2.33–16.2) for all-cause death and MACEs, respectively.

**Table 3:** Adjusted hazard ratios and 95% confidence interval for Hb or RBC by quintiles on outcomes.

**Hb**						***p* for trend^a^**
	**Q1**	**Q2**	**Q3**	**Q4**	**Q5**	
	*n* = 85	*n* = 73	*n* = 85	*n* = 76	*n* = 72	
Median g/dl	10.40	11.80	12.80	13.70	14.90	
All-cause mortality^b^	5.64 (1.89–16.87)	1.83 (0.55–6.14)	1.44 (0.42–4.96)	2.91 (0.93–9.09)	1 (reference)	0.001
MACE^c^	6.15 (2.33–16.2)	2.86 (0.99–8.26)	1.39 (0.44–4.39)	3.28 (1.19–9.08)	1 (reference)	0.000
**RBC**						***p*** **for trend^a^**
	**Q1**	**Q2**	**Q3**	**Q4**	**Q5**	
	*n* = 81	*n* = 76	*n* = 84	*n* = 73	*n* = 77	
Median ×10^12^/L	3.45	3.89	4.23	4.52	4.96	
All-cause mortality^b^	3.0 (1.26–7.17)	1.48 (0.56–3.92)	0.82 (0.30–2.28)	1.00 (0.35–2.28)	1 (reference)	0.002
MACE^c^	2.70 (1.28–5.68)	1.36 (0.58–3.22)	0.77 (0.31–1.90)	0.79 (0.31–2.01)	1 (reference)	0.001

*HRs and 95% CIs were calculated by Cox proportional-hazards regression models using the forward conditional method*.

*HRs, hazard ratios; COPD, chronic obstructive pulmonary disease; LDL-C, low-density lipoprotein-cholesterol; Hb, hemoglobin; Q, quintiles; RBC, red blood cell; MACE, major adverse cardiovascular events (a composite of all-cause death, recurrent rupture, and secondary procedure)*.

a *p for trend was calculated by entering the median value of each category of Hb and RBC quintiles*.

b *Adjusted for age, COPD, hematocrit, cystatin C, urea nitrogen, and LDL-C*.

c *Adjusted for age, COPD, hematocrit, cystatin C, urea nitrogen, creatinine, total cholesterol, and LDL-C*.

### Survival Analysis

**Figure 2:**
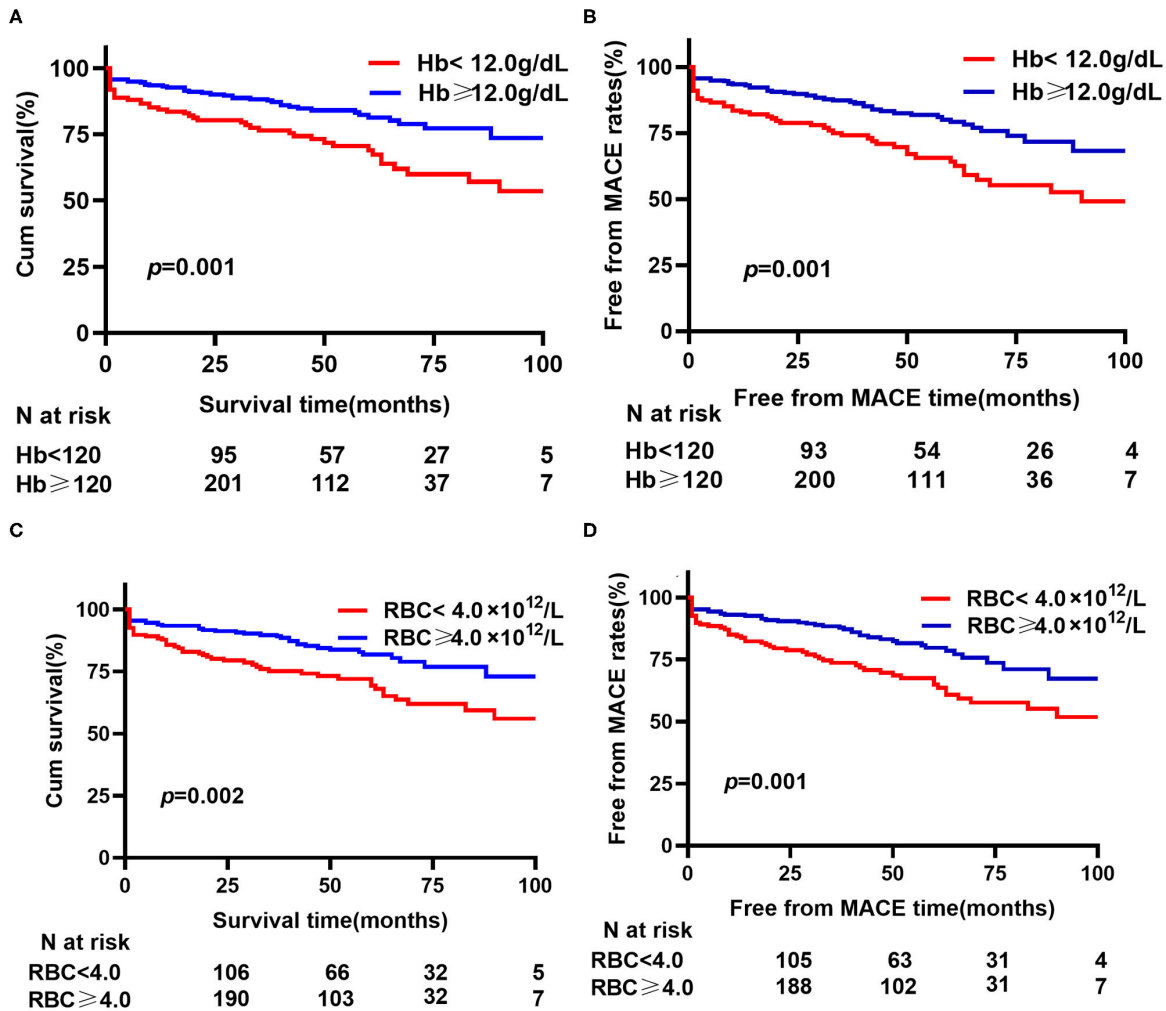
Kaplan–Meier analysis for hemoglobin (Hb) or red blood cell (RBC) on all-cause mortality and MACEs. **(A)** Cumulative all-cause survival rate between different hemoglobin levels. **(B)** Free from MACE rates between different hemoglobin levels. **(C)** Cumulative all-cause survival rate between different red blood cell counts. **(D)** Free from MACE rates between different red blood cell counts. MACE, major adverse cardiovascular events (a composite of all-cause death, recurrent rupture and secondary procedures).

According to Cox multivariate analysis, preoperative Hb [HR (per 1 g/dl) 0.797, 95% CI 0.639–0.918, *p* = 0.002] and LDL-C [HR (per 1 mmol/L) 0.639, 95% CI 0.431–0.948; *p* = 0.026] were independently related to all-cause mortality, following adjustment for age, COPD, hematocrit, urea nitrogen, and cystatin C. For MACEs, when adjusting for age, COPD, hematocrit, cystatin C, urea nitrogen, creatinine, total cholesterol, and LDL-C, preoperative Hb concentration (HR 0.795, 95% CI 0.672–0.871, *p* = 0.000) was significantly associated with MACEs (Supplementary Table 2). Figure 2A illustrates the cumulative all-cause survival rate (log-rank *p* = 0.001) based on Kaplan–Meier analysis, with a significant difference revealed between the two Hb levels; and as indicated in Figure 2B, MACEs were significantly different between them (log-rank *p* = 0.001).

### Prognostic Value of Red Blood Cell

Hb is a conventional biomarker for the oxygen-carrying ability. Given the close association between Hb and RBC, we further estimated the prognostic value of RBC. In the Cox multivariate analysis, RBC [HR 0.457 (per 1 × 10^12^ /L), 95% CI 0.292–0.713, *p* = 0.001] and LDL-C [HR 0.617, 95% CI 0.416–0.914, *p* = 0.016] were independently associated with all-cause mortality, with adjustment for age, COPD, hematocrit, urea nitrogen, and cystatin C. Preoperative RBC [HR 0.419, 95% CI 0.275–0.640, *p* = 0.000] were significantly associated with MACEs, when adjusting for age, COPD, hematocrit, cystatin C, urea nitrogen, creatinine, total cholesterol, and LDL-C (Supplementary Table 2). In Kaplan–Meier analysis, statistical differences were observed for preoperative RBC count levels with regard to all-cause mortality and MACEs (Figures 2C,D).

### Prognostic Value of Anemia

In the Cox multivariate analysis, anemia (HR 3.184 95% CI 1.855–5.465, *p* = 0.000) was significantly related to all-cause mortality, with adjustment for age, COPD, hematocrit, urea nitrogen, cystatin C, and LDL-C. Anemia (HR 2.883, 95% CI 1.738–4.784, *p* = 0.000) and urea nitrogen [HR 1.072 (per 1 mmol/L), 95% CI 1.003–1.146, *p* = 0.042] were significantly associated with MACEs, when adjusting for age, COPD, hematocrit, cystatin C, creatinine, total cholesterol, and LDL-C (Supplementary Table 2). In Kaplan–Meier analysis, anemia was significantly associated with all-cause mortality and MACEs (Supplementary Figure 5).

## Discussion

The results of the present study suggest the following. (1) Preoperative Hb level had a significant effect in predicting the long-term mortality and MACEs of patients who undergo TEVAR due to ABAD, despite adjustments for patient characteristics and other serum parameters. (2) The cut-off values for preoperative Hb, as shown in Supplementary Table 1, were likely to provide an additional option for risk stratification. (3) A higher preoperative Hb may confer a much lower risk of adverse events for the aforementioned patients. (4) Survival rates and free from MACE rates differed enormously between those with preoperative Hb < 12 g/dl and Hb ≥ 12 g/dl. (5) Similar phenomena reiterated in both preoperative RBC count and anemia.

The study presented a significant association between different Hb levels and long-term mortality as well as adverse events for patients who develop ABAD post-TEVAR. A recent study by Gorla et al. (18) investigated the relationship between Hb level and in-hospital mortality. They retrospectively analyzed 144 subjects diagnosed with type B AAS (93 with ABAD). They suggested that postoperative Hb decline and Hb level were identified as independent predictors of in-hospital mortality, after a median of 2.6 years of follow-up; however, no significant difference was shown between different Hb levels for either mortality or other events. The disparity may be explained by the sample number and follow-up time, with ours consisting of a larger sample of patients and a longer follow-up period. Furthermore, because AAS includes acute aortic dissection (AAD), intramural hematoma, and penetrating aortic ulcers, their heterogeneity may also account for the discrepancy.

In accordance with our findings, the Hb concentration is independently associated with outcomes in other cardiovascular diseases, as clarified in previous studies. A multicenter study by Sabatine et al. (21) encompassing 39,922 subjects demonstrated that anemia is a powerful predictor of 30-day major adverse events in those who develop acute coronary syndrome (ACS). Additionally, in patients undergoing percutaneous coronary intervention (22) or coronary artery bypass graft surgery (23), lower Hb levels were independently associated with mortality and non-cardiac events. Moreover, Nuis et al. (24) demonstrated that the prevalence of anemia was higher than 50% in patients who underwent transcatheter aortic valve implantation and that preoperative anemia was significantly related to 1-year mortality. Similar conclusions were documented for patients diagnosed with stroke and heart failure (16, 25). Furthermore, Dakour-Aridi et al. (26) compared 30-day in-hospital adverse events between open and endovascular repair of abdominal aortic aneurysms and suggested lower Hb levels to be associated with in-hospital events for both procedures. The present study added ABAD patients post-TEVAR to the list, especially regarding long-term outcomes.

To examine the credibility and reproductivity of our findings, we investigated not only the relationship between Hb level but also RBC count and outcomes of ABAD patients. Consequently, both Hb and RBC were independently associated with follow-up outcomes. Meanwhile, they both had statistical significance in survival analysis. In the linear trend test, similar results were shown between RBC and Hb. Overall, similar findings were observed for Hb and RBC in our work.

Conventionally, anemia is not included in the risk factors of recent guidelines (3, 27). Ranucci et al. (28) proposed that the low prevalence of anemia as well as the multicollinearity between it and other comorbidities may explain this. Anemia is defined as Hb level < 13 g/dl for men and < 12 g/dl for women according to WHO standard (29), but the definition is different in China due to its specific race and geographical circumstance where the threshold value is 12 g/dl for men and 11 g/dl for women (20). In the present study, we illustrated the independent association between anemia and all-cause mortality or MACEs. Moreover, we displayed cutoff points for both Hb and RBC with regard to all-cause mortality and MACEs (Supplementary Table 1), and a lower value may indicate a higher risk in those who undergo TEVAR. We might provide an alternative choice in risk stratifying preoperative ABAD subjects by cutoff points of 11 g/dl for Hb and 4.05 × 10^12^/L for RBC. Additionally, according to our results, the lowest quintile Hb, compared with the highest, increased the mortality and MACE risk by roughly 6-fold; therefore, this finding might be taken into consideration when assessing the long-term outcomes of ABAD patients post-TEVAR. On the other hand, Sabatine et al. (21) illustrated that ACS patients with Hb values > 17 g/dl had increased mortality than those with 14–15 g/dl (HR 1.79, *p* = 0.007). Nonetheless, as only four of our subjects had a Hb level > 17 g/dl, we did not estimate the effect between them.

The explanations about the relationship between Hb and ABAD may be inferred from the following viewpoints. First, inadequate tissue oxygenation delivery due to anemia may play an important role, which may consequently cause dysfunction in the aorta. Furthermore, Horwich et al. (30) reported that patients who develop anemia are more likely to suffer malnutrition, hemodilution, myocardial ischemia, and renal insufficiency. In fact, these comorbidities are indicators of adverse outcomes in both the long and short terms. Finally, a patient with a lower Hb concentration is more likely to receive blood transfusions. A relatively small retrospective (31) study suggested that transfusion was independently related to 30-day death among patients after elective major vascular surgery, which was verified by following multicenter studies (32, 33). Additionally, Murphy et al. (32) found that transfusion was strongly associated with 1-year mortality postcardiac surgery and that transfusion may be related to race, preoperative anemia, open abdominal aneurysm repair, open bypass, and emergent procedure (33). The mechanism responsible may be a change in stored blood cells functionally and structurally, a process that may lead to an inflammatory response including interleukin-6, lipopolysaccharide-binding protein, and CRP release (34). Thus, unnecessary blood transfusion was not recommended in recent guidelines (35).

Our study had several limitations. First, this was a retrospective single-center study; thus, it should be prudent when expanding the results to subjects in other regions. Second, we could not assess all factors related to all-cause mortality and MACEs. Third, a small number of patients were ultimately lost to follow-up, which may have led to bias in our study. Finally, we only recorded serum parameters at admission, and the values may differ during different periods.

In conclusion, Hb level may serve as a good predictor of long-term adverse events in ABAD patients undergoing TEVAR. A higher Hb concentration may indicate a better outcome, and similar results were found when assessing the prognostic value of RBC and anemia. Hb levels might be taken into consideration when risk stratifying ABAD patients. Nutrition support to increasing preoperative Hb might be one of the additional treatments in patients with Hb levels lower than 12 g/dl and anemia.

## Data Availability Statement

The raw data supporting the conclusions of this article will be made available by the authors, without undue reservation.

## Ethics Statement

The studies involving human participants were reviewed and approved by ethics committee of Xinqiao Hospital. The patients/participants provided their written informed consent to participate in this study.

## Author Contributions

ZG, ZQ, and JJ designed the study. ZA, CH, and LW collected the data. ZG and ZQ analyzed the data and drafted the manuscript. All authors contributed to the article and approved the submitted version.

## Conflict of Interest

The authors declare that the research was conducted in the absence of any commercial or financial relationships that could be construed as a potential conflict of interest.
