# Supplementary material for: Prognostic Value of Preoperative Hemoglobin Levels for Long-Term Outcomes of Acute Type B Aortic Dissection Post-thoracic Endovascular Aortic Repair
Source: Front Cardiovasc Med. 2020 Nov 5;7:588761. doi: 10.3389/fcvm.2020.588761 (PMC7693721; doi:10.3389/fcvm.2020.588761)
Supplement: Supplementary file 1 [file Table_1.DOCX]

**Supplementary Table 1 Cutoff Points and Area under Curves for Hb and RBC on Outcomes**

| **Variables** | **Cut-off Point** | **Sensitivity** | **Specificity** | **Youden Index** | **AUCs** |
| --- | --- | --- | --- | --- | --- |
| **All-cause Mortality** |  |  |  |  |  |
| Hb | 11.05 g/dL | 0.849 | 0.368 | 0.217 | 0.617 |
| RBC | 4.05×10^12^ /L | 0.648 | 0.563 | 0.211 | 0.623 |
| **MACEs** |  |  |  |  |  |
| Hb | 11.15 g/dL | 0.836 | 0.378 | 0.214 | 0.617 |
| RBC | 3.88×10^12^ /L | 0.758 | 0.459 | 0.217 | 0.616 |

RBC: red blood cell count; Hb: hemoglobin; AUC: Area Under Curve; MACE: major adverse cardiovascular events (a composite of all-cause death, recurrent rupture and secondary procedure)

**Supplementary Table 2 Forward Conditional Cox Multivariate Analysis in BAAD Patients Post-TEVAR**

| **All-cause Mortality** | **HR** | **Lower limit** | **Upper limit** | ***p* value** |
| --- | --- | --- | --- | --- |
| **Hb model** |  |  |  |  |
| Hb | 0.797 | 0.693 | 0.918 | 0.002 |
| LDL-C | 0.639 | 0.431 | 0.948 | 0.026 |
| **RBC model** |  |  |  |  |
| RBC | 0.457 | 0.292 | 0.713 | 0.001 |
| LDL-C | 0.617 | 0.416 | 0.914 | 0.016 |
| **Anemia model** |  |  |  |  |
| Anemia | 3.184 | 1.855 | 5.465 | 0.000 |
| **MACEs** |  |  |  |  |
| **Hb model** |  |  |  |  |
| Hb | 0.795 | 0.672 | 0.871 | 0.000 |
| **RBC model** |  |  |  |  |
| RBC | 0.419 | 0.275 | 0.640 | 0.000 |
| **Anemia model** |  |  |  |  |
| Anemia | 2.883 | 1.738 | 4.784 | 0.000 |
| Urea nitrogen | 1.072 | 1.003 | 1.146 | 0.042 |

Hb: hemoglobin; RBC: red blood cell; LDL-C: low density lipoprotein cholesterol; OR: odds ratios; MACEs: all-cause death, recurrent rupture, and secondary procedure

**Supplementary Figure 1：Study Flow Chart of Subjects Enrollment**

Severe heart failure(7), Malignant cancer(4) and Myocardial infarction(4); n=15

loss to follow-up; n=5

n=391

n=396

n=410

445 patients diagnosed with acute Stanford B aortic dissection

Traumatic (21), Marfan syndrome (6), Prior Stanford A dissection (7) and Iatrogenic injury (1); n=35

**Supplementary Figure 3：The Area Under the Receiver Operating Characteristic Curves for Hemoglobin on All-cause Death and MACEs**

***p*=0.428**

***p*=0.344**

***p*=1.000**

**Hb≥12.0 g/dL**

**Supplementary Figure 2：In-hospital Mortality, Secondary Procedure and Recurrent Rupture Rates between Different Hemoglobin Levels**

**Hb<12.0 g/dL**

**Rates (%)**

**Recurrent**

**Ruptures**

**Secondary**

**Procedures**

**In-hospital**

**Mortality**

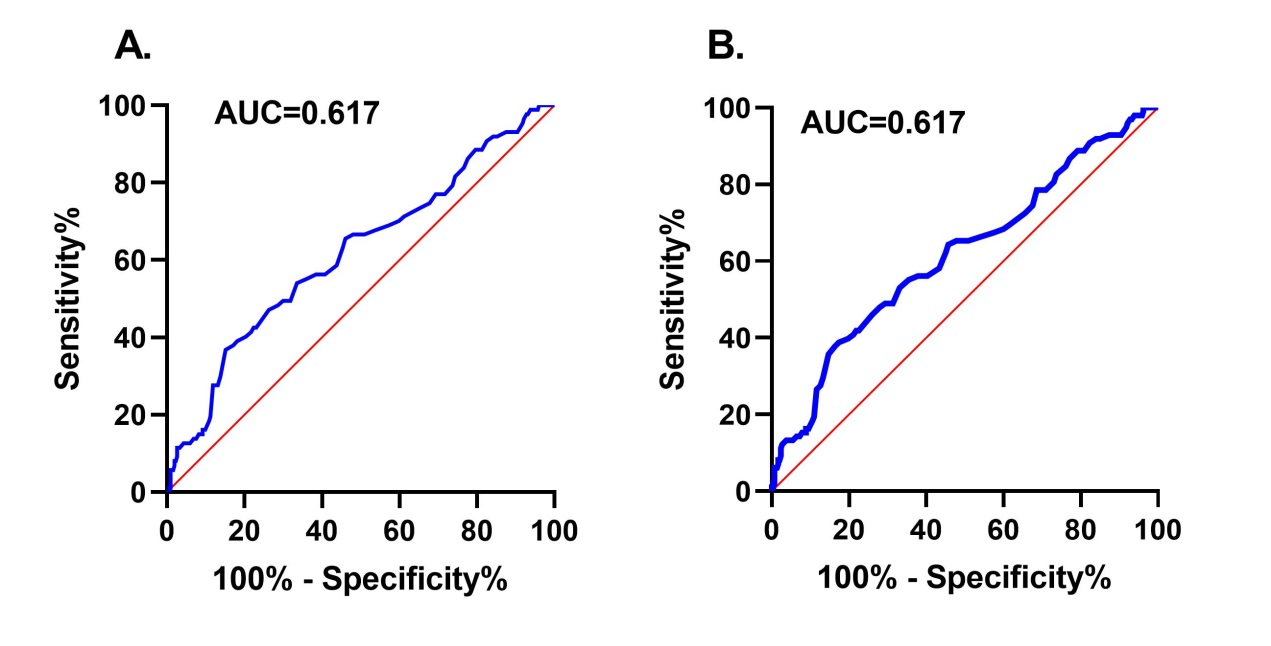


**(B)**

**(A)**

(A): The area under the receiver operating characteristic curve for Hemoglobin on all-cause death

(B): The area under the receiver operating characteristic curve for Hemoglobin on MACEs;

MACE: major adverse cardiovascular events (a composite of all-cause death, recurrent rupture and secondary procedures)

**Supplementary Figure 4：The Area Under the Receiver Operating Characteristic Curves for RBC on All-cause Death and MACEs**

**(A)**

**(B)**


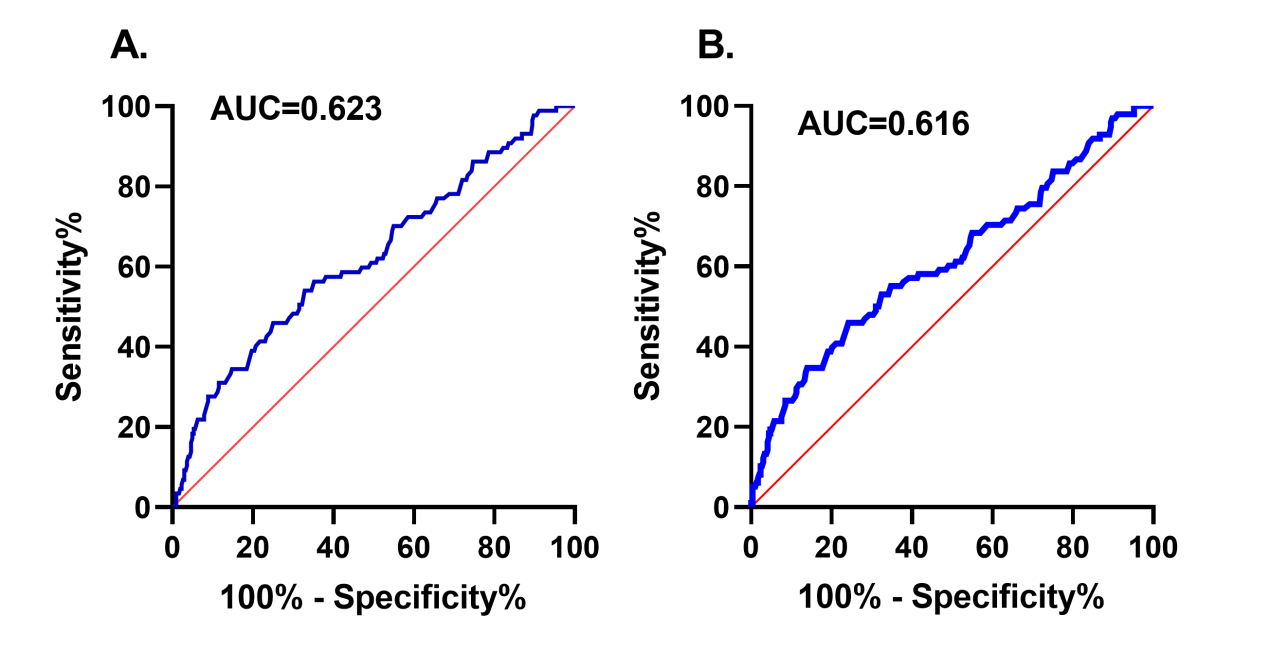


(A): The area under the receiver operating characteristic curve for red blood cell count on all-cause death

(B): The area under the receiver operating characteristic curve for red blood cell count on MACE;

MACE: major adverse cardiovascular events (a composite of all-cause death, recurrent rupture and secondary procedures)

**Supplementary Figure 5: Kaplan-Meier Analysis for Anemia on All-cause Mortality and MACEs**

(A): Cumulative all-cause survival rate in anemia; (B): Free from MACE rates in anemia.
